# Supplementary material for: The causal role of circulating inflammatory markers in osteoporosis: a bidirectional Mendelian randomized study
Source: Front Immunol. 2024 Jul 18;15:1412298. doi: 10.3389/fimmu.2024.1412298 (PMC11291241; doi:10.3389/fimmu.2024.1412298)
Supplement: Supplementary file 1 [file DataSheet_1.zip › supplementary material/CIM-OSTEOPOROSIS /GCST90274843.csv_forest.pdf]

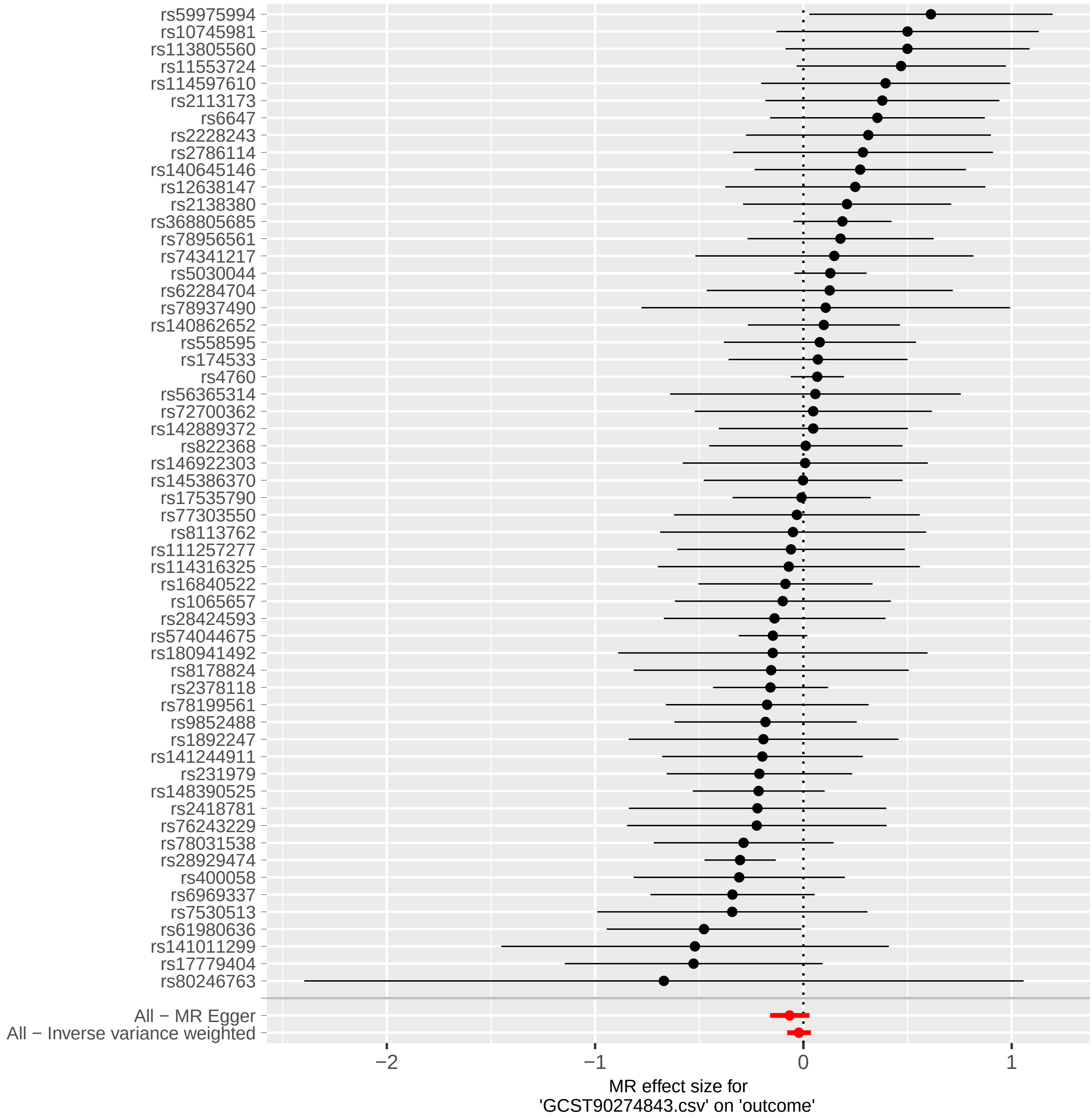
rs59975994
rs10745981
rs113805560
rs11553724
rs114597610
rs2113173
rs6647
rs2228243
rs2786114
rs140645146
rs12638147
rs2138380
rs368805685
rs78956561
rs74341217
rs5030044
rs62284704
rs78937490
rs140862652
rs558595
rs174533
rs4760
rs56365314
rs72700362
rs142889372
rs822368
rs146922303
rs145386370
rs17535790
rs77303550
rs8113762
rs111257277
rs114316325
rs16840522
rs1065657
rs28424593
rs574044675
rs180941492
rs8178824
rs2378118
rs78199561
rs9852488
rs1892247
rs141244911
rs231979
rs148390525
rs2418781
rs76243229
rs78031538
rs28929474
rs400058
rs6969337
rs7530513
rs61980636
rs141011299
rs17779404
rs80246763
All − MR Egger
All − Inverse variance weighted
−2
−1
0
1
MR effect size for
'GCST90274843.csv' on 'outcome'
